# A methodology for using Lambda phages as a proxy for pathogen transmission in hospitals

**DOI:** 10.1016/j.jhin.2023.01.004

**Published:** 2023-01-20

**Authors:** K.B. Burke, B.A. Berryhill, R. Garcia, D.A. Goldberg, J.A. Manuel, P.R. Gannon, B.R. Levin, C.S. Kraft, J.M. Mumma

**Affiliations:** a Department of Biology, Emory University, Atlanta, GA, USA; b Division of Infectious Diseases, Department of Medicine, Emory University School of Medicine, Atlanta, GA, USA; c Program in Microbiology and Molecular Genetics, Graduate Division of Biological and Biomedical Sciences, Laney Graduate School, Emory University, Atlanta, GA, USA; d Department of Pathology and Laboratory Medicine, Emory University School of Medicine, Atlanta, GA, USA

**Keywords:** Hospital-acquired infection, Bacteriophage, Contamination, Infection prevention, Lambda phage, Healthcare workers

## Abstract

**Background:**

One major concern in hospitalized patients is acquiring infections from pathogens borne on surfaces, patients, and healthcare workers (HCWs). Fundamental to controlling healthcare-associated infections is identifying the sources of pathogens, monitoring the processes responsible for their transmission, and evaluating the efficacy of the procedures employed for restricting their transmission.

**Aim:**

To present a method using the bacteriophage Lambda (λ) to achieve these ends.

**Methods:**

Defined densities of multiple genetically marked λ phages were inoculated at known hotspots for contamination on high-fidelity mannequins. HCWs then entered a pre-sanitized simulated hospital room and performed a series of patient care tasks on the mannequins. Sampling occurred on the scrubs and hands of the HCWs, as well as previously defined high-touch surfaces in hospital rooms. Following sampling, the rooms were decontaminated using procedures demonstrated to be effective. Following the conclusion of the simulation, the samples were tested for the presence, identity, and densities of these λ phages.

**Findings:**

The data generated enabled the determination of the sources and magnitude of contamination caused by the breakdown of established infection prevention practices by HCWs. This technique enabled the standardized tracking of multiple contaminants during a single episode of patient care. Unlike other biological surrogates, λ phages are susceptible to common hospital disinfectants, and allow for a more accurate evaluation of pathogen transmission.

**Conclusion:**

Whereas our application of these methods focused on healthcare-associated infections and the role of HCW behaviours in their spread, these methods could be employed for identifying the sources and sites of microbial contamination in other settings.

## Introduction

One of the greatest risks of being hospitalized is acquiring infections from pathogenic microbes in treatment environments (e.g. catheter-associated urinary tract infections or catheter-associated bloodstream infections) [1]. These pathogens can be unknowingly transmitted by healthcare workers (HCWs) to patients, thereby contributing to patient morbidity and mortality [2]. Previous studies using mathematical models have shown that one solution to reducing the incidence of hospital-acquired infections is to increase the efficacy of measures for preventing the transmission of pathogens from HCWs to patients (e.g. hand hygiene and barrier precautions) [3–5]. Central to designing, implementing, and evaluating these measures is elucidating the sources of the pathogens responsible for infections and the pathways for transmitting those pathogens to patients.

As a patient care environment can harbour different sources of pathogens and abounds in opportunities for their transmission, there is a need to trace multiple transmission pathways simultaneously [6]. Existing approaches using non-biological or biological surrogates for pathogens do not satisfy the need for in-situ evaluation of transmission events. Non-biological surrogates, such as fluorochrome-tagged body fluids, can be used to simulate different sources of contamination simultaneously but not the susceptibility of pathogens to common antiseptics (e.g. alcohol-based hand rub [7]). Approaches using biological surrogates, including live viruses and viral DNA markers, are also limited. Viruses, such as bacteriophage MS2, are susceptible to both antiseptics and disinfectants, but can only be used to trace a single source of contamination at a time [8]. Viral DNA markers, such as cauliflower mosaic virus DNA or silica nanoparticles with encapsulated DNA, can produce multiple unique markers but are unaffected by antiseptics and certain disinfectants (e.g. quaternary ammonium compounds [8–10]). Thus, a method is needed for tracing multiple transmission pathways simultaneously during patient care, each of which can be counteracted by common infection prevention and control measures, such as performing hand hygiene with alcohol-based hand rub or disinfecting environmental surfaces.

This report presents a methodology and preliminary results using genetically marked variants of the bacteriophage Lambda (λ) as a harmless surrogate for pathogen transmission. We then validate this method in a naturalistic setting by contaminating different surfaces prior to simulated patient care in a hospital environment.

## Methods

### Reagents and equipment

The following reagents and equipment were used: LuriaeBertani (LB) Broth Miller (Difco, BD, Franklin Lakes, NJ, USA, Product #244620), LB Plates (Difco, Product #244510), 0.65% Agar LB soft agar (Difco, Product #214030), LaboPlast Spray Bottle with Pump Vaporizor (Bürkle, Bad Bellingen, Germany, Product #10216–888), Self-contained 0.85% Saline Swab (Hardy Diagnostic, Santa Maria, CA, USA, Product #SRK35), RNase Away Reagent (Invitrogen, Carlsbad, CA, USA, Product #10328–011), DNA Away (Molecular BioProducts, California, USA), 70% ethanol solution (Decon Labs, King of Prussia, PA, USA, Product #2716), Ruler, Disinfecting Wipes (Lysol, NJ, USA, Product #3168342. Active Ingredient: Alkyl dimethyl benzyl ammonium chloride 0.26%). Whirl-Pak (Madison Industries, Chicago, USA, Product #B01542), Sterile Saline Wipes (Hygea, Doral, FL, USA, Product #C22370), Phusion Blood Direct PCR (polymerase chain reaction) Master Mix (Thermo Fisher, Vilnius, Lithuania, Product #F-175L), O’Gene Ruler DNA Ladder (Thermo Scientific, Waltham, MA, USA, Product #SM1563), 10,000× GelRed Nucleic Acid Strain (Biotium, Fremont, CA, USA, Product #41003), 70% ethanol v/v Purell Advanced Green Certified Gel (Gojo Industries, Inc., Akron, OH, USA, Product #1903–02), Sani-Cloth germicidal disposable wipes (Professional Disposables International, Orangeburg, NY, USA, Product #Q55172), saline wound flush spray (MediChoice, Owens & Minor, Mechanicsville, VA, USA, Product #SWF071), and Aloetouch Protect patient wipes (Medline, Morthfield, IL, USA, Product #PF66411).

### Strains

*Escherichia coli* strain C was acquired from Marie-Agnès Petit from INRAe, France. Bacteriophage λ (λ^Temp^), Bacteriophage λ^Chl^, Bacteriophage λ^Kan^, and Bacteriophage λ^Vir^ were obtained from Maros Pleska at The Rockefeller University, New York, NY, USA.

### Lysate preparation

1e^5^ plaque-forming units (PFU)/mL of each phage were cultured with 1e^7^ cfu/mL log-phase *E. coli* in 10 mL of LB broth grown at 37 °C. These cultures were grown with shaking for 6 h before being centrifuged and filtered through a 0.22 μm filter to generate sterile, high-titre lysates of each of the four bacteriophages. These lysates were serially diluted and plated on lawns of *E. coli* to determine viral titers.

### Phage distribution

Ten millilitres of 1e^8^ PFU/mL of each type of λ phage lysate was loaded into their respective spray bottles. The spray bottles were stored at 4 C and transported on ice. Each bottle was primed by spraying five times into a waste container. Immediately after priming, each lysate was sprayed with one pump from a distance of 10 cm on to two target sites on two high-fidelity mannequins (patients 1 and 2): λ^Temp^ on patient 1’s wound, λ^Kan^ on patient 1’s stool, λ^Chl^ on patient 2’s groin, and λ^Vir^ on patient 2’s stool. The spray dried clear such that target sites were unidentifiable to the naked eye. Contamination occurred no earlier than 30 min before the start of the simulation.

### Phage recovery

**Figure 1.**
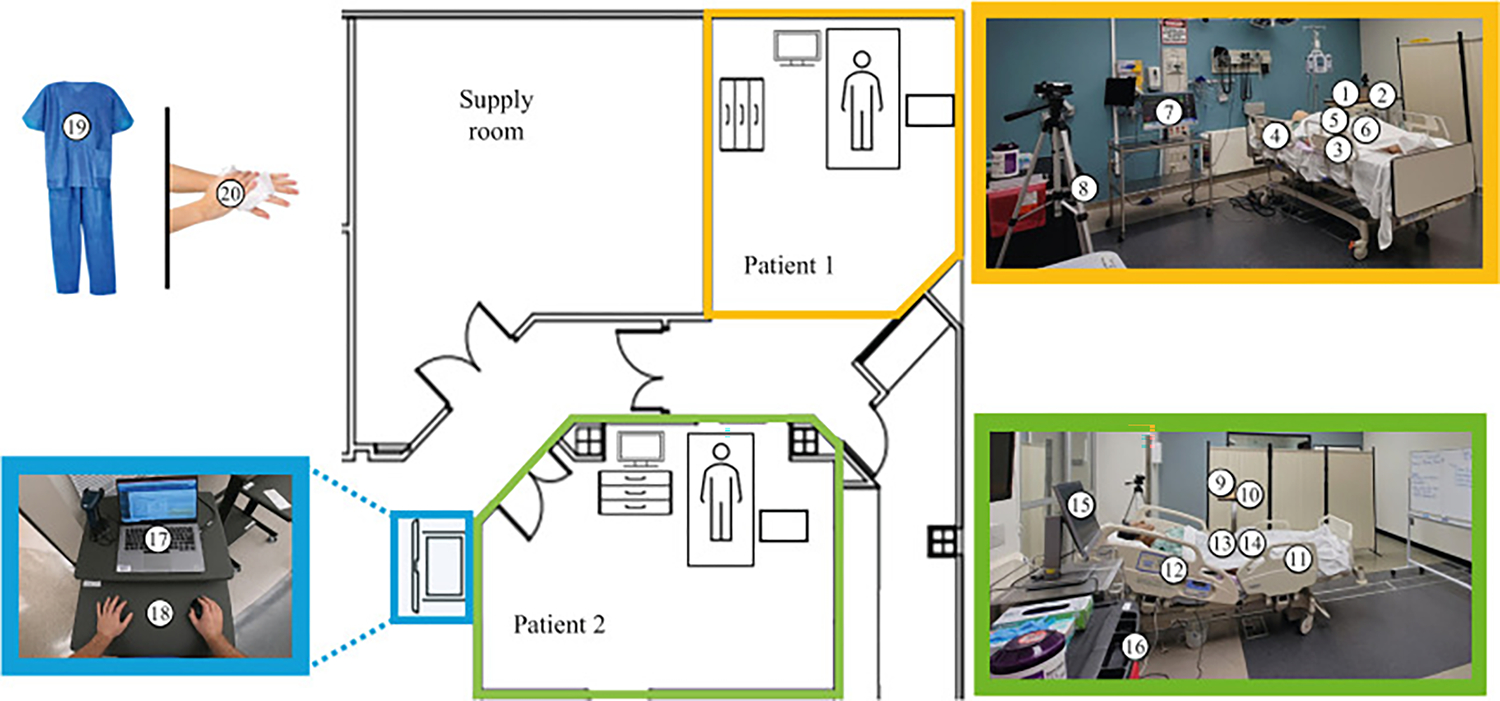
Sampling design. Diagram of the simulated hospital environment with the sampling sites numbered. (Yellow inset) One simulated hospital room with sampling sites marked. (Green inset) Second simulated hospital room with sampling sites marked. (Blue inset) Two sampling sites located on the healthcare worker’s mobile workstation.

Immediately after the simulation, HCW hands were sampled by applying a saline hand wipe around both hands and forearms. The saline hand wipe was placed into a conical tube for storage. HCWs then placed their disposable scrubs into a Whirl-Pak for storage. High-touch surfaces (see Table I and Figure 1) were sampled with self-contained 0.85% saline swabs [11]; each swab was removed from the saline, and the surface was swabbed in a progressive back-and-forth motion until the entire surface became damp from the saline. The swab was then returned to the saline solution. To liberate the phage from the saline hand wipe, the wipe was squeezed to remove the excess liquid and the extracted solution was used for testing. To recover phage from the disposable scrubs, 300 mL of deionized water was added to the bags that contained the scrubs, shaken vigorously to ensure that scrubs were fully saturated, and excess liquid was poured into a Falcon tube for later processing.

### Site decontamination

After the phage recovery phase, a liberal coating of 70% ethanol was applied to all surfaces upon which the HCW could have interacted. This alcohol was removed, and this cleaning step was repeated with DNA Away, RNase Away, and Lysol.

### Phage identification and quantification

The samples were tested for the presence, identity, and densities of the four Lambda phages. Phage identification was performed by PCR, using Thermo Scientific’s Phusion Blood Direct PCR Master Mix. Products were visualized on a 1% agarose/TAE gel with Biotium’s 10,000× GelRed Nucleic Acid Stain. Band sizes of 800 bp were called λ^Temp^ or λ^Vir^, 1500 bp called λ^Chl^, and 1900 bp called λ^Kan^. Temperate phage (λ^Temp^) and the virulent mutant (λ^Vir^) were distinguished during the phage quantification step (Supplementary Figure A1).

The serum resistance lipoprotein (*bor*) gene (Gene ID: 2703532, NCBI) of the Lambda phages was amplified by PCR using the following primers designed in PrimerBLAST (NCBI): forward (borRG1Fw) 5′-GCTCTGCGTGATGATGTTGC-3′ and reverse (borRG1Rv) 5′-GCAGAGAAGTTCCCCGTCAG-3′. Using the double-layer soft agar method, LB soft agar overlays containing 0.1 mL of a fully turbid *E. coli* overnight were prepared and allowed to harden. Serially diluted saline recovery solution (0.01 mL) was spotted on the overlay at four densities. These plates were grown overnight at 37 °C, and plaques were enumerated the next day. Based on the turbidity of the plaques, λ^Vir^ was distinguished from the other three temperate forms (see Supplementary Figure A2).

### High-fidelity simulations

Across two pre-sanitized simulated hospital rooms, defined densities of genetically marked Lambda phages were sprayed on to two target sites on two mannequins, as described previously. After contamination, HCWs, comprising registered nurses from the emergency department, intensive care units, or medical/surgical floors, performed four tasks for two patients over the next hour. For each patient, two of the four tasks required the HCW to interact with a source of contamination: changing a dressing on a simulated stage-4 pressure injury (λ^Temp^ on patient 1’s wound), toileting a patient with a bedpan (λ^Kan^ on patient 1’s stool), inserting a Foley catheter (λ^Chl^ on patient 2’s groin), and collecting a stool specimen (λ^Vir^ on patient 2’s stool). HCWs knew that contamination may be present in the simulation but were unaware of the location of contamination and sampling sites. HCWs wore disposable scrubs over their clothing. Personal protective equipment (e.g. gloves and gowns) and medical-grade products for cleaning patients and disinfectants were available to every HCW during the simulation (see ‘Susceptibility to disinfecting and cleaning’). Additionally, HCWs documented their work in an electronic medical record, accessed on a mobile ‘workstation on wheels’ (WoW). As the simulations were conducted as part of a larger study of 45 HCWs, we present the results of a subset of ten randomly selected simulations performed in the manner described above.

## Results

### Method calibrations

#### Susceptibility to disinfecting and cleaning

**Figure 2.**
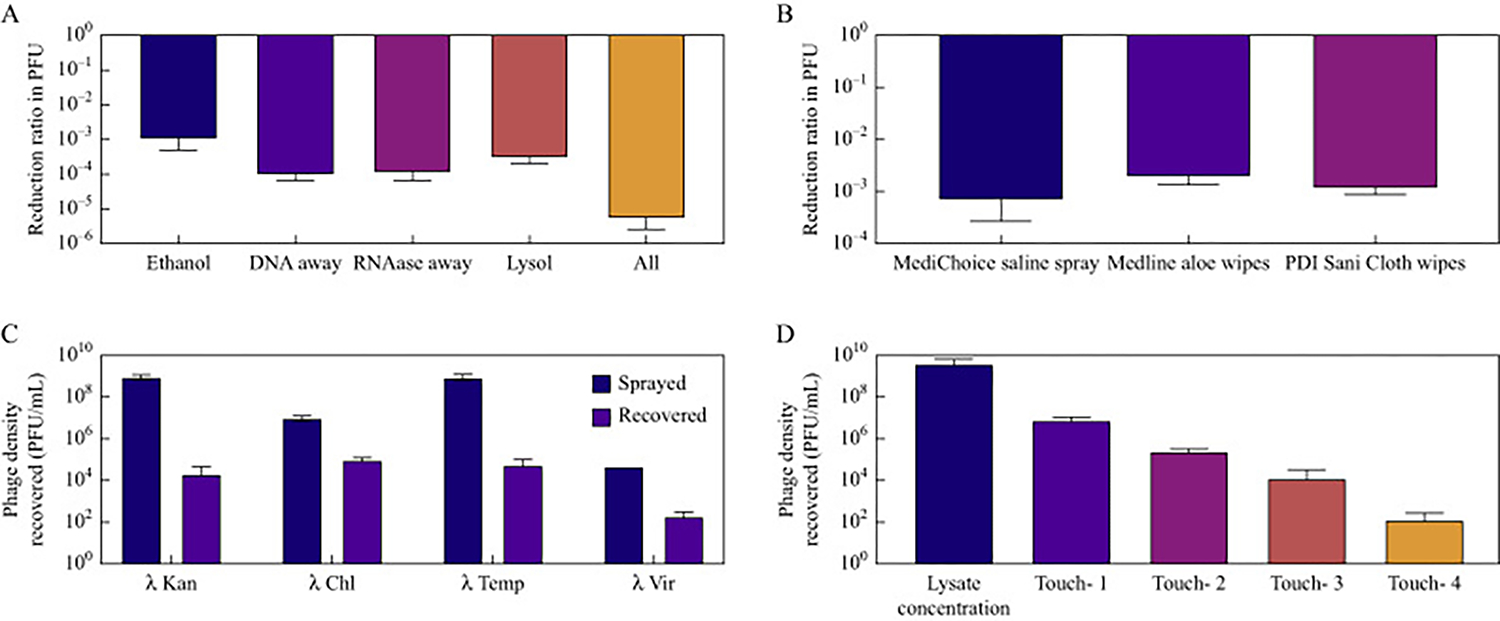
Phage recovery experiments. Experimental results of the effect of antiseptics, time, and surface transfer in sprayed bacteriophage recovery. The density of each type of λ phage lysate that was used in the experiments was 1e^8^ PFU/mL. (A) Reduction rate of phage density on surfaces after exposure to products used to decontaminate simulations. (B) Reduction rate of phage density after exposure to cleaning and disinfecting products used by HCWs during simulations (C) Phage density of the lysate (blue) and the recovery 2 h post spray on a surface (purple) at room temperature. (D) Phage density of the lysate (blue), the density of the residual phage left on the sprayed surface (purple) after being touched and transferred to a new surface, and the density of phage recovered from the new surface (pink).

We tested the ability of different products to eliminate phages and report changes in PFU (Figure 2A). Products used to decontaminate surfaces after simulations (ethanol 70%, DNA Away, RNase Away, and Lysol wipes) individually reduced the amount of bacteriophage by a minimum of 3 log_10_ and a maximum of 4 log_10._ The combination of these products in our decontamination protocol eliminated the phage so that it was undetectable by PCR. There was no difference in the reduction ratio to the disinfectants among the phages used. The lack of detectability by PCR demonstrates the effectiveness of our decontamination protocol after simulations. Moreover, λ phages were reduced by alcohol (in a concentration frequently used in hand rubs), unlike other biological proxies that produce multiple markers [8–10].

During simulations, HCWs used products for cleaning patients (Medline AloeTouch Protect patient wipes and MediChoice saline spray for wound care) and disinfection (Purell hand rub containing ethanol 70% and PDI Super Sani-Cloth disinfecting wipes containing quaternary ammonium and isopropyl alcohol). The patient wipes, saline spray, and disinfecting wipes reduced the amount of bacteriophage by a minimum of 2 log_10_ and a maximum of 3 log_10_ (Figure 2B).

#### Decay over time

We estimated the rate of decay of each λ phage variant on a surface over time and from the changes in the density of PFU over a given period (Figure 2C). The bacteriophages were sprayed on a surface and allowed to sit for 2 h. The time of 2 h was more than the allotted time that the bacteriophages would be sitting on a surface in the medical simulations. After the 2 h, the surface was then swabbed and plated for recovery. There was a 2e3 log_10_ rate of decay in recoverable PFU over time. This same rate of decay between λ phages differentiates it from other bacteriophages used in medical simulations, such as Phi6 and MS2 [6].

#### Efficiency of sampling

To determine the efficiency of sampling, phages were sprayed at a known density on a defined area which was then sampled via swabbing. The recovered swabs were then plated on a lawn of *E. coli* for PFU estimation. The efficiency of swabbing recovery was estimated to be >80%.

#### Spray bottle variability

The variability between sprays was evaluated in terms of volume and surface area from a given distance away. To ensure that the spray bottle was distributing a consistent and measurable volume, a weigh boat was placed in an analytical scale and tared. The weigh boat was sprayed with one full squeeze from a spray bottle, and the weight of the sample was recorded. It was assumed that the weight in grams was equivalent to the volume of phage sprayed. The average volume sprayed per pump was 0.215 mL (0.00269). The phage suspension was sprayed from different distances of 5, 10, and 15 cm above the bench, to modify the surface area covered. The spray was performed perpendicular to the bench, and the recorded diameter of the spray was defined as the outermost dark ring measured. The diameters for 5, 10, and 15 cm away were 65 (±1), 80 (±5), and 105 (±10) mm, respectively. Thus, we selected 10 cm as the distance at which to spray the phage on the initial sites of contamination.

#### Ability to spread to multiple surfaces

To determine the ability of our technique to track phages across multiple surfaces touched by gloved hands, we sprayed a surface with the marked λ phages, which were then touched, spread to other places via subsequent touches, and then sampled. Four subsequent touches were performed from initial lysate, with each new transfer beginning at the previously touched location. Reported are the changes in PFU between touched surfaces (Figure 2D).After subsequent touching, phage was recovered from the initial inoculation site as well as all the sites where the phage was transferred via subsequent touch. The initial inoculation side saw a 1–3 log_10_ reduction in PFU in comparison to the lysate used. The side that was subsequently touched, which initially had no phage, sawa2–4log_10_ reduction in PFU in comparison to the lysate used. This 2 log_10_ reduction was seen between each subsequent touch performed. When comparing the initial inoculation side to the subsequently touched sides, there was less than a 1 log_10_ difference in PFU that were recovered across all lambda phage strains.

#### PCR sensitivity

The sensitivity of the PCR detection method was evaluated by serially diluting a stock of the marked λ phages at known PFU/mL, or number of infectious viruses, and performing PCRs at each dilution (Table I). All strains were 100% recoverable through the 1e^2^ PFU/mL, 58% recoverable (7/12) at 1e^1^ PFU/mL, 58% recoverable (7/12) at 1e^0^ PFU/mL, and 75% recoverable (9/12) at 1e^−1^ estimated PFU/mL. The difference in recovery rate is likely due to chance because of the small sample volume (1 μL) used in the PCR processing (Table II).

### Method validation with high-fidelity simulations

For each simulation and λ variant, six binary outcomes were measured: (1) whether transmission of a phage occurred within a patient room, (2) between patient rooms, (3) to the nurse, (4) to the WoW, (5) to a critical site on a patient, or (6) to another (non-critical) patient site (Table III). A total of 42 transmission events was observed across the ten simulations, with a median of 5 transmission events (range: 1–8) occurring per simulation. Transmission events occurred most frequently within patient rooms (29% of all events), to the nurse (19%), at similar frequencies between patient rooms (14%), to the WoW (14%), or to a critical site on a patient (14%), and least frequently to another (non-critical) site on a patient (10%). Whether transmission events resulted from a single or a series of touches could not be determined.

**Figure 3.**
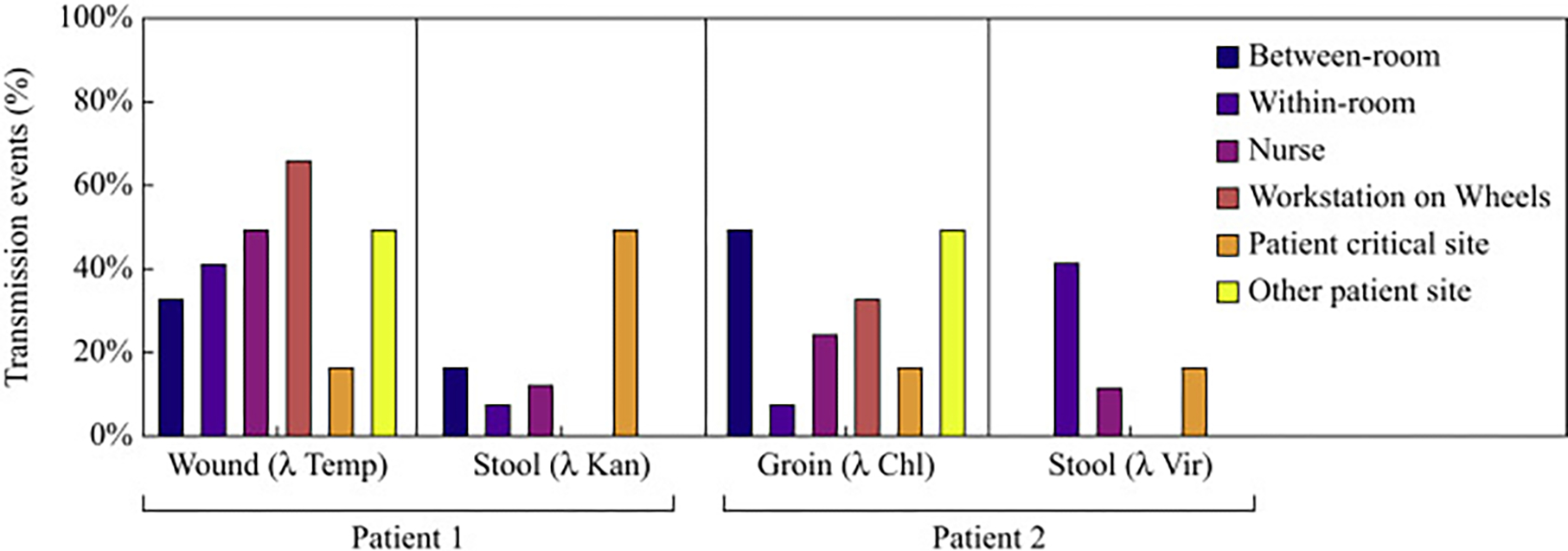
Percentage of transmission event types originating from contamination sources. Data from ten randomly selected simulations are shown. Bars in dark blue are transmissions between the two patient rooms, in orange are transmissions within the patient’s own room, in grey recovery from the nurse, in yellow recovery from the workstation on wheels, and in light blue recovery of phage from a critical site on a patient. Bars of the same colour sum to 100% across the four sources of contamination.

The four sources of contamination varied in their involvement in transmission events; 50% of all transmission events originated from patient 1’s wound (λTemp), 32% from patient 2’s groin (λChl), 21% from patient 2’s stool (λVir), and 18% from patient 1’s stool (λKan). Regarding involvement in types of transmission event (Table III), Figure 3 shows the percentage of each type of transmission event originating from each source. All six types of transmission events can be traced back to at least two of the four sources of contamination. At least half of all transmission events to the nurse (50%) or to the WoW (67%) originated from patient 1’s wound alone. Most transmission events within patient rooms (83%) originated from either patient 1’s wound or patient 2’s stool, and similarly, most transmissions between patient rooms (83%) originated from either patient 1’s wound or patient 2’s groin. Patient 1’s stool alone contributed to half of the transmission events to a critical site on a patient. Lastly, phage was infrequently detected on a non-critical patient site (i.e. surfaces on patients where the contaminated stool was applied), often originating from another source of contamination on that same patient (e.g. wound or groin). Apart from transmission events, a median of 2 (range: 1–3) of the 4 sources of contamination per simulation was positive for the phage with which they were inoculated, despite HCWs cleaning each source (e.g. using patient wipes or saline flush during wound care).

## Discussion

This report describes a method for using variants of bacteriophage λ as surrogates for pathogen transmission. These variants contain unique genetic markers, which permit the identification of the source and transmission path of each phage via PCR. The calibration experiments showed that the effect of disinfectants, decay after 2 h, and transfer recovery had no difference among the four phages used in this project. The calibrations allow for the interchangeable ability of these viruses if used in different simulated environments and differentiate them from previous methods used to track contaminations.

To validate the transmission dynamics of λ phages in a naturalistic setting, each phage was inoculated on to a surface in a simulated hospital environment prior to simulated patientcare. The patterns of dissemination observed in these simulations resemble those that occur during actual patient care; in the simulations, most transmission events involved the movement of phage from a patient to a high-touch surface in that patient’s room (e.g. to the bedrails, bedside table, or vital signs monitor). In clinical practice, frequent contact between an HCW, their patient, and the patient’s immediate environment rapidly colonizes high-touch surfaces with a patient’s own flora [12]. Transmission to the HCW (e.g. clothing or hands), which contributes to transmission between patients or their rooms, was also relatively more common [10]. Among the least frequent events, but most concerning, was the transmission of phage to a critical site on a patient. Compared to other types of transmission events, transmission to a patient critical site is relatively less common during actual patient care but increases the risk of a patient developing an infection [2,12].

An advantage of the present method is that it allows for transmission events to be traced back to different sources of contamination (e.g. patient care tasks). Demonstrating that the four phages were differentially involved in transmission events in the simulations provides further validation of their dynamics in naturalistic settings; for example, phage from patient 1’s wound (a simulated stage-4 pressure injury) was the variant most frequently involved in transmission events, particularly to surfaces on the WoW or the nurse. Wound care is considered a high-contact patient care task, which creates opportunities for pathogens to be transferred to HCW hands or clothing [13]. Consequently, in 2019, the Centers for Disease Control and Prevention began recommending the use of gowns and gloves when performing wound care in skilled nursing facilities (where multidrug-resistant organism (MDRO) transmission is common), regardless of a nursing home resident’s MDRO colonization or infection status [13]. In accordance with this recommendation, the present method identified wound care as a frequent contributor to transmission events, particularly those that may disseminate pathogens between patients.

As calibration experiments demonstrated the equivalence of the λ variants, differences in how each variant was disseminated in simulated patient care reflect other factors, such as characteristics of tasks (e.g. amount of patient contact) or the infection prevention practices of HCWs. Consequently, this method is useful for identifying the sources of contamination that contribute most to transmission events, where sources could be located simultaneously in different rooms (e.g. to examine MDRO transmission between patients [10]), body sites of a patient (e.g. to assess the role of endogenous microbes in hospital-acquired infections [12]), or surfaces on an HCW (e.g. to evaluate the contribution of different items of personal protective equipment to HCW self-contamination during doffing [7]). Lastly, unlike similar surrogates, λ phages are susceptible to widely used disinfectants such as alcohol-based hand rub and surface disinfectants, so the effect of infection prevention practices on transmission may also be evaluated [9].

The present method is not without limitations. Unlike other surrogates, like fluorescent tracers, live viruses do not provide immediate feedback about the occurrence of transmission events. Although less useful for rapidly training HCWs in infection prevention practices, the ability to simulate multiple sources of pathogens simultaneously and realistically lends itself to rigorous research or quality improvement efforts (e.g. evaluating the effectiveness of the training programme) [7]. Lastly, the number of available l variants is limited, but any similar phages with detectable variation at one gene could be employed for future efforts.

In conclusion, four variants of the bacteriophage λ were used as surrogates for pathogens to track transmission events in a simulated hospital environment. Analyses of the results from ten simulations in which HCWs performed common patient care tasks revealed that λ phages can identify the sources of pathogen transmission and assess their differential involvement in transmission events within and between patient rooms, to mobile surfaces, and to critical sites on patients. Whereas existing approaches using non-biological or biological surrogates for pathogens have succeeded in simulating multiple sources of contamination, but not the susceptibility of pathogens to common disinfectants (e.g. alcohol-based hand rub), the present method is notable for achieving both. The applicability of the present method is broad but is particularly relevant to understanding the sources and pathways of MDRO transmission in healthcare settings.

## Supplementary Material

Supplementary Material

## Figures and Tables

**Table I:** Surfaces sampled in each simulation

Type of surface	Sampling surface	Number in Figure 1

High touch	Bedside table	1, 2 (patient 1); 9, 10 (patient 2)
	Bedrails	3 (patient 1); 11 (patient 2)
	Bedrail buttons	4 (patient 1); 12 (patient 2)
	Vital signs monitor	7 (patient 1); 15 (patient 2)
	Supply cart	8 (patient 1); 16 (patient 2)

Healthcare worker	Scrubs	19
	Bare hands	20

Workstation on wheels	Keyboard	17
	Table	18

Patient critical site	Wound	5
	Groin	13

Other patient site	Site of stool contamination	6 (patient 1); 14 (patient 2)

**Table II:** Polymerase chain reaction (PCR) sensitive for the detection of λ phage

Estimated PFU/mL	λ *Kan*^a^	λ *Chl*^a^	λ *Temp*^a^	λ *Vir*^a^	Positive PCR (%)
E^9^	XXX	XXX	XXX	XXX	100
E^8^	XXX	XXX	XXX	XXX	100
E^7^	XXX	XXX	XXX	XXX	100
E^6^	XXX	XXX	XXX	XXX	100
E^5^	XXX	XXX	XXX	XXX	100
E^4^	XXX	XXX	XXX	XXX	100
E^3^	XXX	XXX	XXX	XXX	100
E^2^	XXX	XXX	XXX	XXX	100
E^1^	XX	XXX	X	X	58
E^0^	X	XX	XX	XX	58
E^−1^	XX	XXX	XXX	X	75

a An X is assigned for every positive PCR amplification out of three replicas.

**Table III:** Definition of transmission events

Transmission event	Definition
Between-room	A phage from one patient was recovered from at least one high-touch surface in the other patient’s room.
Within-room	A phage from one patient was recovered from at least one high-touch surface in the same patient’s room.
Nurse	A phage was recovered from at least one surface on the nurse.
Workstation on wheels	A phage was recovered from at least one surface on the workstation on wheels.
Patient critical site	A phage was recovered from a critical site on a patient, excluding phage introduced to the site as part of the simulation.
Other patient site	A phage was recovered from a non-critical patient site.
