# Supplementary material for: A methodology for using Lambda phages as a proxy for pathogen transmission in hospitals
Source: J Hosp Infect. Author manuscript; Available in PMC 2024 Jan 18. (PMC10795484; doi:10.1016/j.jhin.2023.01.004)
Supplement: Supplementary Material [file NIHMS1955996-supplement-Supplementary_Material.docx]

**Supplementary Information**

**
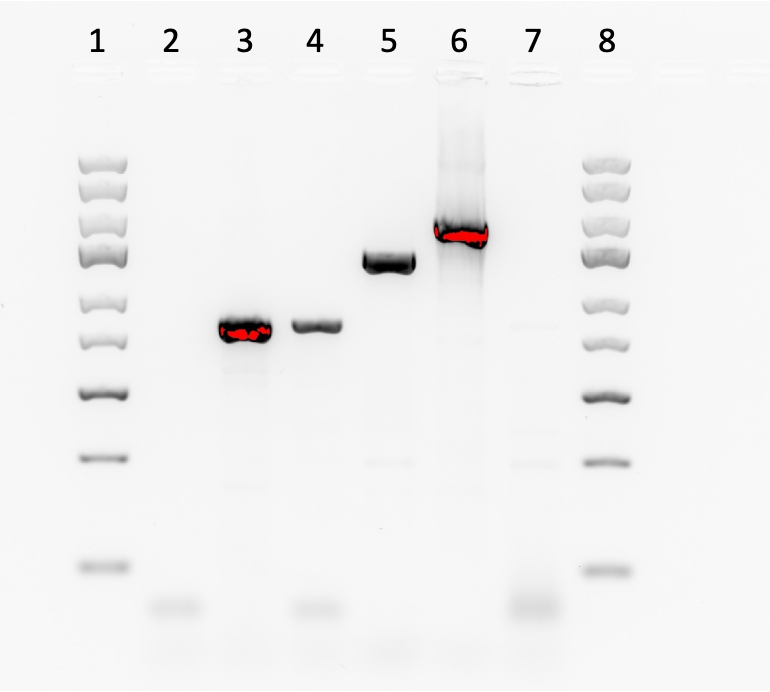
**

**Supplemental Figure 1. PCR Differentiation of Lambda phage variants.** Shown from left to right are the (1) Standard Ladder, (2) a water control, (3) λ^Temp^, (4) λ^Vir^, (5) λ^Chl^, (6) λ^Kan^, an (7) *Escherichia coli* bacterial host control, and a (8) O’GeneRuler Express DNA Ladder (5kb).

**
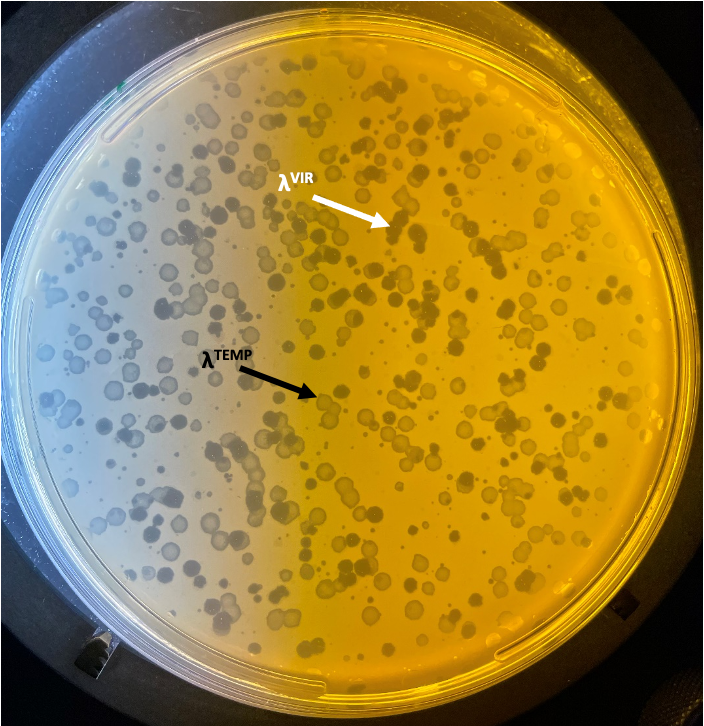
**

**Supplemental Figure 2. Plaque morphology difference in** λ**^Temp^ and** λ**^Vir^.** Shown is a double-layer soft agar lawn of *E. coli* C containing both λ^Temp^ (turbid plaques) and λ^Vir^ (clear plaques).
